# Periodontitis and type 2 diabetes among women with previous gestational diabetes: epidemiological and immunological aspects in a follow-up of three years

**DOI:** 10.1590/1678-77572016-0367

**Published:** 2017

**Authors:** Rafael Paschoal ESTEVES LIMA, Luis Otávio Miranda COTA, Tarcília Aparecida SILVA, Sheila Cavalca CORTELLI, José Roberto CORTELLI, Fernando Oliveira COSTA

**Affiliations:** 1 Universidade Federal de Minas Gerais, Faculdade de Odontologia, Departamento de Periodontologia e Patologia, Belo Horizonte, MG, Brasil.; 2 Universidade de Taubaté, Centro de Pesquisa Periodontal, Taubaté, SP, Brasil.

**Keywords:** Gestational diabetes, Diabetes mellitus, Diabetes mellitus, type 2, Periodontitis, Pregnant women

## Abstract

Periodontitis can contribute to the development of insulin resistance. Gestational diabetes is a risk factor for type 2 diabetes. Therefore, periodontitis, when associated with gestational diabetes, could increase the risk for the development of type 2 diabetes after pregnancy.

**Objective:**

The aim of this study was to verify the incidence on the development of type 2 diabetes in women with previous gestational diabetes with and without periodontitis after a three-year time interval.

**Material and Methods:**

Initial sample of this follow-up study consisted of 90 women diagnosed with gestational diabetes who underwent periodontal examination. After three years, 49 women were subjected to new periodontal examination and biological, behavioral, and social data of interest were collected. Additionally, the quantification of the C-reactive protein in blood samples was performed. Fasting glucose and glycated hemoglobin levels were requested. Saliva samples were collected for quantification of interleukin 6 and 10, tumor necrosis factor α, matrix metalloproteinase 2 and 9.

**Results:**

The incidence of type 2 diabetes mellitus was 18.4% and of periodontitis was 10.2%. There was no significant difference in the incidence of type 2 diabetes mellitus among women with and without periodontitis. It was observed impact of C-reactive protein in the development of type 2 diabetes mellitus. However, it was not observed impact of periodontitis on the development of type 2 diabetes mellitus among women with previous gestational diabetes.

**Conclusions:**

It was not observed impact of periodontitis on the development of type 2 diabetes among women with previous gestational diabetes. The impact of C-reactive protein in the development of type 2 diabetes mellitus highlights the importance of an inflammatory process in the diabetes pathogenesis.

## Introduction

Periodontitis is an infectious inflammatory condition of periodontal tissues characterized by loss of tooth support^1^. The production of inflammatory mediators in the pathogenesis of the disease called the attention to the systemic impact of periodontitis and its potential association with other conditions^13^.

Diabetes mellitus (DM) comprises a group of disorders characterized by high blood glucose levels, and it is considered an important risk factor for periodontitis^2^. Alterations in the immuno-inflammatory response of individuals with DM can influence the prevalence, extension, and severity of periodontitis. On the other hand, the periodontal inflammatory process can contribute to a condition of insulin resistance, with impact on the glycemic control and manifestation of DM. The dissemination of bacteria and their products by periodontitis can induce a systemic inflammatory state that can initiate and propagate insulin resistance. High levels of inflammatory mediators, such as tumor necrosis factor alpha (TNF-α), interleukin 6 (IL-6), and C-reactive protein (CRP), might contribute to an increase in insulin resistance^24^. Additionally, periodontal bacteria can translocate to the liver, inhibit the insulin signaling, and result in decreased glycogen synthesis^17^. Recent systematic reviews have demonstrated that periodontal therapy can positively affect the control of DM^11^ ^,^ ^22^.

Gestational DM (GDM) is a hyperglycemic status that initiates during gestation. It presents significant associated complications and high morbidity. An expressive risk for the development of DM type-2 (DM-2) was reported among women with GDM. Therefore, the diagnosis of GDM constitutes an opportunity for early intervention of DM-2^2^.

Based on the possibility that periodontitis can contribute to the development of insulin resistance, periodontitis, when present in women with GDM, could also increase the risk for the development of DM-2 after the gestational period. To our knowledge, only one study evaluated the impact of periodontitis on the incidence of DM-2 among women with previous GDM, pointing out that women with a history of GDM and periodontitis have impaired glucose metabolism^29^. Therefore, additional studies are necessary to better address this potential association.

The aim of the present follow-up study was to verify the incidence on the development of type 2 diabetes in women with previous gestational diabetes with and without periodontitis after a three-year time interval, and to quantify CRP in the blood as well as TNF-α, IL-6, interleukin 10 (IL-10), matrix metalloproteinase 2 (MMP-2), and matrix metalloproteinase 9 (MMP-9) in the saliva.

## Material and methods

### Sampling strategy

The sample of the present follow-up study initially comprised a cohort of 90 women previously diagnosed with GDM from a previous case-control study that investigated the association between periodontitis and GDM^13^. These women received prenatal care in the Odete Valadares Maternity Hospital, in Belo Horizonte city – Brazil, from February 2010 to November 2011, period when they were diagnosed with GDM.

Subsequently, these 90 women were invited to participate in the present study by phone or mail contact. From this total, 39 women could not be located and two refused to participate. Therefore, the final sample comprised 49 women with a previous diagnosis of GDM.

The established inclusion criteria were the presence of ≥12 natural teeth and absence of contraindications for the periodontal examination. The exclusion criteria included antibiotic or periodontal therapy three months prior to clinical examination and positive human immunodeficiency virus infection serology.

The present study was approved by the Ethics Research Committee from the Federal University of Minas Gerais (CAAE 28708814.6.0000.5149). Participants were informed about the study and signed an informed consent form.

### Sociodemographic characteristics

Social and demographic data were collected for each participant through structured questionnaires. We collected data regarding age, marital status, educational level, gestational period and delivery date, parity, smoking habits, and first degree relatives with DM. Regarding smoking, women were classified as smokers, former smokers, and non-smokers^25^.

### Medical data

Weight and height of participants were recorded and body mass index (BMI) was calculated. According to BMI, women were classified as underweight, normal weight, overweight, or obese^27^.

Fasting glucose test and glycated hemoglobin levels were collected for each participant. When exams showed altered glycemic levels, they were performed again to confirm the diagnosis of DM-2. Values higher than 125 mg/dl for fasting glucose and 6.4% for glycated hemoglobin were considered positive for the diagnosis of DM-2. Pre-diabetes was diagnosed as the presence of values higher than 99 mg/dl for fasting glucose and 5.6% for glycated hemoglobin, according to the American Diabetes Association^2^ (2014). Sample was divided in two groups according to glycemic levels: a) normal fasting glucose group (NFG); b) altered fasting glucose group (AFG). Subsequently, they were subdivided in three groups according to glycemic diagnosis: normal, pre-diabetes, and DM-2.

Additionally, we requested a blood test for assessing the CRP levels. The CRP level less than 5 mg/l was considered normal. Values greater than or equal to 5 mg/l were considered abnormal^20^.

### Periodontal clinical examination

Participants underwent a periodontal examination during gestation in a previous study^13^, determined to be the baseline examination (T0), when parameters of bleeding on probing (BOP), probing depth (PD), and clinical attachment level (CAL) were evaluated. At T0, the prevalence of periodontitis was 40% among women with GDM^13^.

Participants underwent a new periodontal examination after a period of approximately three years after delivery (34.5±6.4 months), determined to be the final examination (T1). Periodontal exams comprised circumferential probing with the recording of periodontal parameters at four sites *per* tooth (distal, mesial, buccal, and lingual) with a manual probe (UNC-15, Hu-Friedy, Chicago, IL). Periodontal examinations were performed by a single periodontist (R.P.E.L.), trained and calibrated, responsible for the initial examination of the participants. Intra-examiner agreement for all clinical periodontal parameters of interest, both at T0 and T1, showed *kappa* values higher than 0.90.

All collected data were recorded and evaluated for each participant in order to define study groups. The following exclusion criteria were also adopted during periodontal examination: third molars, teeth whose cementum-enamel junction was impossible to determine, teeth with gingival morphology alterations, teeth with extensive caries lesions, teeth with iatrogenic restorative procedures, excessive calculus presence.

The criteria for periodontitis definition was the presence of ≥4 teeth having ≥1 sites with PD ≥4 mm and CAL ≥3 mm associated with BOP^15^. Periodontitis was classified in relation to extension, according to the number of affected sites: 30% of sites (localized), and >30% of sites (generalized). Periodontitis was also classified in relation to severity, according to the amount of attachment loss: 1-2 mm (slight form), 3-4 mm (moderate form), and ≥ 5 mm (severe form)^3^.

### Salivary examination

Stimulated whole saliva sample collections were performed to quantify the levels of IL-6, IL-10, MMP-2, MMP-9, and TNF-α. The collection of saliva was performed considering the period of two hours after the last meal. For the stimulus of the salivary flow, the participants chewed the hyperboloid for five minutes and the saliva produced was collected on graph tubes. The saliva sample was kept in a cooler with ice and it was transported immediately to the laboratory where the total volume was registered and centrifuged at 3000 rpm for 15 minutes at 4°C. The volume was measured with a micropipette. After centrifuging, the saliva was diluted in the proportion of 1:1 in PBS solution (0.4 mM NaCl and 10 mM NaPO_4_) containing protease inhibitors (0.1 mM phenyl methyl sulfonyl fluoride, 0.1 mM benzethonium chloride, 10 mM EDTA and 0.01 mg/mL aprotinin A). The solution was homogenized, distributed in aliquots, and froze at - 80°C to perform the analysis by ELISA. The concentrations of the cytokines IL-10, MMP-2, MMP-9, and TNF-α in the saliva samples were determined by the sandwich technique using the DuoSet Kit (R&D Systems, Minneapolis, MN, USA). Detection limits were from 31.2 to 1000 pg/mL for TNF-α, 390-12500 pg/mL for MMP-9/TIMP2, 125-4000 pg/mL for MMP-2/TIMP2, and 125-2000 pg/mL for IL-10. The quantification of IL-6 was performed using the human IL-6 Kit and its quantikine (R&D Systems, Minneapolis, MN), with detection limits from 0.156 to 10 pg/mL. Techniques were performed according to the manufacturer’s specifications. The concentrations were expressed in pg/mL. The concentration of total protein was used to correct the cytokine value for each sample. The corrected values were expressed as pg/mg.

### Statistical analysis

Initially, a descriptive analysis of the sample and a comparison of periodontal status between T0 and T1 were performed. For this purpose, the Wilcoxon test was used for quantitative variables and the McNemar or Stuart-Maxwell (for more than two levels of comparison) were used for categorical variables.

For the univariate analysis, considering the NFG and AFG groups, the Mann-Whitney and Kruskal-Wallis tests were used for quantitative variables, and the Chi-squared or Fisher exact testes were used for categorical variables. Subsequently, the influence of biological, behavioral, and social variables in alterations of fasting glucose and glycated hemoglobin exams was analyzed through a multinomial logistic regression. A 25% significance level in the univariate analysis, as well as the biological plausibility, was adopted for the selection of variables to enter the models. All collected data were stored in a database (S1 Dataset), and all analyses were performed by means of statistical software (R version 3.0.1, R Foundation for Statistical Computing, Vienna, Austria). Results were considered significant for a probability lower than 5% (p<0.05).

## Results

We present the characteristics of the sample according to biological, behavioral, and social variables for NFG and AFG groups in Table 1. We observed significant differences regarding mean BMI and glycemic values. Similar results were observed in the analysis of the biological, behavioral, and social data according to fasting glucose diagnosis at T1. There was a significant difference between the groups regarding mean BMI (p=0.014). The incidence of DM-2 was 18.4%.

**Table 1:** Characteristics of the sample in relation to variables of interest according to glycemic control at T1

Variables	Total sample (n=49)	Glycemic control		p
		NFG (n=34; 69.4%)	AFG (n=15; 30.6%)	
Age in years (±)	35.3 ± 5.1	34.9 ± 4.9	36.1 ± 5.4	0.317*
Marital status (%)				0.765**
with companion	41 (83.7%)	29 (85.3%)	12 (80.0%)	
without companion	7 (14.3%)	4 (11.8%)	3 (20.0%)	
Other	1 (2.0%)	1 (2.9%)	0 (0.0%)	
Educational level (%)				0.482**
≤ 8 years	22 (44.9%)	14 (41.2%)	8 (53.3%)	
From 9 to 12 years	25 (51.0%)	19 (55.9%)	6 (40.0%)	
≥ 13 years	2 (4.1%)	1 (2.9%)	1 (6.7%)	
Parity (±)	2.4 (1.3)	2.4 (1.1)	2.2 (1.9)	0.136*
Time since delivery in months (±)	34.5 ± 6.4	34.1 ± 6.3	35.6 ± 6.8	0.467*
Smoking habits (%)				
smoker	2 (4.1%)	2 (5.9%)	0 (0.0%)	1.000**
former smoker	8 (16.3%)	6 (17.2%)	2 (13.3%)	0.702**
Diabetes (%)	4 (8.2%)	0 (0.0%)	4 (28.6%)	0.005**
Family history of diabetes (%)	29 (59.2)	18 (52.9)	11 (73.3)	0.221**
BMI (%)				0.070**
adequate	13 (26.5)	12 (35.3)	1 (6.7)	
overweight	16 (32.7)	11 (32.4)	5 (33.3)	
obesity	20 (40.8)	11 (32.4)	9 (60.0)	
BMI (mean)	30.3 (7.5)	28.6 (6.8)	33.9 (7.7)	0.014*
Fasting glucose (mean)	103 (33.6)	87.4 (7.5)	138.7 (42.2)	0.000*
Glycated hemoglobin (mean)	5.5 (1.1)	5.0 (1.0)	6.9 (1.0)	0.000*

*Mann-Whitney test; **Chi-squared test; NFG = normal fasting glucose; AFG = altered fasting glucose

Table 2 shows the quantification of biochemical variables for NFG and AFG groups. We described CRP categorically and quantitatively. Important differences in the levels of CRP were observed between the groups. Approximately 53% of the AFG women demonstrated changes in CRP levels, while in the NFG group this percentage was 23.5% (p=0.040). When CRP was quantitatively evaluated, a higher average was observed in the AFG (p=0.077). There was not any significant difference between groups for IL-6, IL-10, MMP-2, MMP-9, and TNF-α values. Similar results were observed for the analysis of the concentrations of inflammatory mediators in blood and saliva according to endocrine diagnosis. There was a difference between the groups regarding CRP described categorically.

**Table 2:** Characteristics of the sample in relation to the concentration of inflammatory mediators in blood and saliva according to glycemic control

Variable	Glycemic control		p
	NFG (n=34; 69.4%)	AFG (n=15; 30.6%)	
CRP			0.040*
Normal	26 (76.5)	7 (46.7)	
Altered	8 (23.5)	8 (53.3)	
CRP (mg/l)	5.4 (10.4)	7.8 (7.9)	0.077**
IL-10 (pg/mg)	24.8 (61.5)	31.2 (68.0)	0.723**
MMP-9 (pg/mg)	1356.4 (1053.1)	1136.4 (986.0)	0.641**
MMP-2 (pg/mg)	126.8 (150.7)	156.8 (163.2)	0.494**
IL-6 (pg/mg)	55.3 (93.4)	41.6 (73.3)	0.956**
TNF-α (pg/mg)	32.8 (90.1)	27.8 (58.8)	0.312**

*Chi-squared test; **Mann-Whitney test; NFG = normal fasting glucose; AFG = altered fasting glucose; CRP = c-reactive protein; IL-6 = interleukin 6; IL-10 = interleukin 10; MMP-2 = matrix metalloproteinase 2; MMP-9 = matrix metalloproteinase 9; TNF-α = tumor necrosis fator alpha

We present periodontal status at T0 and T1 for NFG and AFG groups in Table 3. At T1, the frequency of periodontitis was higher in AFG group (46.7%) when compared with NFG group (35.3%). However, this difference was not statistically significant, whereas 38.8% of the sample was diagnosed with periodontitis at T1. The incidence of periodontitis in AFG was 20%. It was observed a significant difference regarding the number of teeth from T0 to T1 in the sample. However, this difference was not present when groups were compared. Women in the AFG group presented a higher percentage of sites with BOP and higher PD. From T0 to T1, women in the AFG group presented a significant increase in the number of sites with BOP and PD 4 mm and CAL ≥3 mm. Characteristics of the sample concerning periodontal status at T0 and T1 are presented in Table 4, according to the endocrine diagnosis. There was no significant difference regarding the frequency of periodontitis, as well as periodontal parameters, among the groups in both examinations.

**Table 3:** Characteristics of the sample in relation to periodontal variables according to glycemic control at T0 and T1

Variables	Total sample n=49 (%)	Glycemic control		p
		NFG n=34 (69.4%)	AFG n=15 (30.6%)	
Presence of periodontitis (%)				
T0	20 (40.8)	16 (47.1)	4 (26.7)	0.221*
T1	19 (38.8)	12 (35.3)	7 (46.7)	0.451*
p	0.763***	0.157***	0.083***	
Present teeth (±)				
T0	27.5 (3.0)	27.3 (3.2)	27.9 (1.9)	0.635**
T1	27.1 (2.9)	26.9 (3.3)	27.7 (2.2)	0.515**
p	0.002****	0.000****	0.427****	
Extension of periodontitis (%)				
Localized T0	20 (40.8)	16 (47.1)	4 (26.7)	0.221*
Localized T1	17 (34.7)	11 (32.4)	6 (40.0)	0.741*
p	0.404***	0.096***	0.317***	
Extension of periodontitis (%)				
Generalized T0	0 (0.0)	0 (0.0)	0 (0.0)	-
Generalized T1	2 (4.1)	1 (2.9)	1 (6.7)	0.489*
p	0.157***	0.317***	0.317***	
Severity of periodontitis (%)				
Moderate T0	1 (2.0)	1 (2.9)	0 (0.0)	1.000*
Moderate T1	3 (6.1)	2 (5.9)	1 (6.7)	1.000*
p	0.317***	0.563***	0.317***	
Severity of periodontitis (%)				
Advanced T0	19 (38.8)	15 (44.1)	4 (26.7)	0.341*
Advanced T1	16 (32.7)	10 (29.4)	6 (40.0)	0.515*
p	0.366***	0.095***	0.157***	
Sites with BOP (%)				
T0	22.9 (15.9)	22.6 (15.7)	23.6 (17.0)	0.905**
T1	32.5 (21.7)	29.8 (20.6)	38.5 (23.7)	0.121**
p	0.001****	0.047****	0.004****	
Sites with CAL ≥3 mm and PD 4 mm (%)				
T0				
T1	2.8 (3.3)	2.9 (2.8)	2.7 (4.3)	0.314**
	4.0 (5.7)	3.7 (5.1)	4.8 (7.1)	0.315**
p	0.067****	0.3757****	0.009****	
Sites with CAL ≥3 mm and PD 5-6 mm (%)				
T0	2.2 (3.3)	2.2 (3.1)	1.8 (2.5)	0.740**
T1	2.1 (5.7)	2.1 (4.2)	2.3 (3.9)	0.456**
p	0.876****	0.868****	0.683****	
Sites with CAL ≥3 mm and PD ≥7 mm (%)				
T0	0.1 (0.4)	0.2 (0.6)	0.1 (0.4)	0.178**
T1	0.1 (0.5)	0 (0.0)	0.1 (0.3)	0.635**
p	1.000****	0.371****	1.000****	

*Chi-squared test; **Mann-Whitney test; ***McNemar test; ****Wilcoxon test; CAL = clinical attachment level; PD = probing depth; BOP = bleeding on probing; NFG = normal fasting glucose; AFG = altered fasting glucose

**Table 4:** Characteristics of the sample in relation to periodontal variables according to endocrine diagnosis at T0 and T1

Variables	Endocrine diagnosis			p
	Normal n=34 (69.4%)	Pre-diabetes n=6 (12.2%)	Type 2 diabetes n=9 (18.4%)	
Presence of periodontitis (%)				
T0	16 (47.1)	2 (33.3)	2 (22.2)	0.451*
T1	12 (35.3)	4 (66.7)	3 (33.3)	0.316*
p	0.157***	0.157***	0.317***	
Present teeth (±)				
T0	27.3 (3.2)	28.7 (1.2)	27.4 (2.2)	0.548**
T1	26.9 (3.3)	28.3 (1.4)	27.2 (2.6)	0.626**
p	0.000****	1.000****	1.000****	
Extension of periodontitis (%)				
Localized T0	16 (47.1)	2 (33.3)	2 (22.2)	0.451**
Localized T1	11 (32.4)	4 (66.7)	2 (22.2)	0.221**
p	0.096***	0.157***	1.000***	
Extension of periodontitis (%)				
Generalized T0	0 (0.0)	0 (0.0)	0 (0.0)	
Generalized T1	1 (2.9)	0 (0.0)	1 (11.1)	0.523**
p	0.317***	-	0.317***	
Severity of periodontitis (%)				
Moderate T0	1 (2.9)	0 (0.0)	0 (0.0)	1.000 **
Moderate T1	2 (5.9)	1 (16.7)	0 (0.0)	0.401**
p	0.563***	0.317***	-	
Severity of periodontitis (%)				
Advanced T0	15 (44.1)	2 (33.3)	2 (22.2)	0.547**
Advanced T1	10 (29.4)	3 (50.0)	3 (33.3)	0.647**
P	0.095***	0.317***	0.317***	
Sites with BOP (%)				
T0	22.6 (15.7)	27.6 (16.7)	20.9 (17.7)	0.646**
T1	29.8 (20.6)	42.9 (19.0)	35.6 (27.1)	0.292**
p	0.047****	0.094****	0.024****	
Sites with CAL ≥3 mm and PD 4 mm (%)				
T0	2.9 (2.8)	2.6 (3.5)	2.7 (5.1)	0.590**
T1	3.7 (5.1)	4.4 (2.6)	5.1 (9.2)	0.224**
p	0.375****	0.062****	0.090****	
Sites with CAL ≥3 mm and PD 5-6 mm (%)				
T0	2.2 (3.1)	2.5 (3.2)	1.3 (1.9)	0.776**
T1	2.1 (4.2)	3.1 (3.7)	1.8 (4.1)	0.793**
p	0.868****	0.787****	1.000****	
Sites with CAL ≥3 mm and PD ≥7 mm (%)				
T0				
T1	0.2 (0.6)	0 (0.0)	0 (0.0)	0.391**
	0.1 (0.4)	0 (0.0)	0.1 (0.3)	0.191**
p	0.371****	-	1.000****	

*Chi-squared test; **Kruskal-Wallis test; ***McNemar test; ****Wilcoxon test; CAL = clinical attachment level; PD = probing depth; BOP = bleeding on probing

We present changes in periodontal status from T0 to T1, for both AFG and NFG groups, and according to the endocrine diagnosis, in Table 5. In the AFG group, the percentage of women who develop periodontitis between T0 and T1 was higher. In addition, women in the AFG group presented worse periodontal parameters from T0 to T1. The frequency of periodontitis at both T0 and T1, or only at T1, was higher among women diagnosed with pre-diabetes, although not statistically significant. Women diagnosed with DM-2 presented a higher increase in PD from T0 to T1.

**Table 5:** Characteristics of the sample in relation to periodontal variables according to glycemic control and endocrine diagnosis from T0 to T1

Variable	Fasting glucose		p	Endocrine diagnosis			p
	NFG	AFG		Normal	Pre-diabetes	Type 2 diabetes	
	n=34 (69.4%)	n=15 (30.6%)		n=34 (69.4%)	n=6 (12.2%)	n=9 (18.4%)	
Occurrence of periodontitis (T1–T0)			0.202*				0.339*
No T0 - No T1	16 (47.1)	8 (53.3)		16 (47.1)	2 (33.3)	6 (66.7)	
No T0 - Yes T1	2 (5.9)	3 (20.0)		2 (5.9)	2 (33.3)	1 (11.1)	
Yes T0 - Yes T1	10 (29.4)	4 (26.7)		10 (29.4)	2 (33.3)	2 (22.2)	
Yes T0 - No T1	6 (17.6)	0 (0.0)		6 (17.6)	0 (0.0)	0 (0.0)	
Mean of sites with BOP (%) (T1–T0)	7.2 (19.3)	15.0 (14.4)	0.121**	7.2 (19.3)	15.3 (17.3)	14.7 (13.3)	0.292**
Sites with CAL ≥3 mm and PD 4 mm (%) (T1–T0)	0.8 (4.0)	2.1 (3.5)	0.092**	0.8 (4.0)	1.8 (1.7)	2.4 (4.4)	0.224**
Sites with CAL ≥3 mm and PD 5-6 mm (%) (T1–T0)	- 0.1 (2.8)	0.6 (2.7)	0.532**	- 0.1 (2.8)	0.7 (2.4)	0.5 (3.1)	0.793**
Sites with CAL ≥3 mm and PD ≥7 mm 4 mm (%) (T1–T0)	- 0.1 (0.2)	0.1 (0.3)	0.111**	- 0.1 (0.2)	0 (0.0)	0.1 (0.3)	0.191**

*Stuart-Maxwell test; **Wilcoxon test; CAL = clinical attachment level; PD = probing depth; BOP = bleeding on probing; NFG = normal fasting glucose; AFG = altered fasting glucose

In the final multivariate logistic regression model, only CRP remained as a significant variable with glycemic control (OR 1.31; 95% CI=1.03-13.45; p=0.046). Similarly, the multinomial logistic regression model (considering the endocrine diagnosis) retained only CRP as a significant variable for DM-2 (OR 6.50; p=0.022).

We also performed all analyses using glycated hemoglobin to establish the endocrine diagnosis. Similar results were observed using fasting glucose diagnosis. It is important to highlight that we observed a high and significant agreement between fasting glucose and glycated hemoglobin examinations (kappa 0.841).

## Discussion

The biological plausibility that the inflammatory process induced by periodontitis could contribute to insulin resistance, and DM-2 development can also be applied to GDM. The number of studies that evaluated the relationship between periodontitis and GDM is reduced^4^ ^,^ ^5^ ^,^ ^9^ ^,^ ^13^ ^,^ ^28^. Moreover, findings from these studies are controversial. Some studies^5^ ^,^ ^28^ demonstrated that periodontitis was more frequent among women with GDM compared with women without GDM. However, other studies^4^ ^,^ ^9^ ^,^ ^13^ did not identify differences in the frequency of periodontitis among women with and without GDM. Important methodological differences, such as sample size and diagnostic criteria for periodontitis and GDM, do exist and difficult the comparisons of the results.

Findings from the present study demonstrated that the frequency of periodontitis was not higher among women with previous history of GDM that developed DM-2. A previous study^29^ evaluated the association between periodontitis and the DM-2 development among women with previous GDM. In this study from Xiong, et al.^29^ (2013), women with GDM and periodontitis were only compared with women without both conditions and no significant differences were found in the most glycemic indexes evaluated. In addition, the study presented lower sample and short follow-up period as well as it used glucose averages. In our understanding, it can interfere with the results, hindering the comparison with the present study. In addition, our results did not show any impact of the levels of IL-6, IL-10, MMP-2, MMP-9, and TNF-α in the saliva on DM-2 or pre-diabetes.

Some studies found that the treatment of periodontitis does not impact the metabolic control of individuals with DM-2^7^ ^,^ ^12^. However, some systematic reviews and meta-analysis^11^ ^,^ ^22^ ^,^ ^24^, confirmed a beneficial effect of periodontal therapy on glycemic levels among individuals with DM-2.

Thus, it is important to emphasize that the fact that the sample included in our study presents, mostly, a more localized periodontitis could explain some divergences of results. We can also hypothesize that in a sample with a more generalized periodontitis, consequently with a high systemic inflammation, the impact on insulin resistance and DM-2 development could be more evident. Although not statistically significant, individuals with altered glycemic status presented a worsening in periodontal status between both examinations. Findings showed an increase in the frequency, extension, and severity of periodontitis as well as in the percent of sites with BOP, PD, and CAL.

One study^18^ evaluated the effect of tooth extraction on glycemic control of individuals with DM-2, demonstrating that individuals undergoing dental treatment showed a significant reduction in glycated hemoglobin levels compared with individuals who did not receive any treatment. This finding emphasizes the potential systemic impact of the inflammatory-infectious process of the oral cavity in the metabolic control and the development of an insulin resistance.

Similar results observed for inflammatory mediators were previously described. One systematic review^8^ concluded that diabetic and non-diabetic individuals had similar levels of IL-6 in the saliva, suggesting a lack of association between IL-6 from an inflammatory process in the oral cavity and DM-2. In another study, the expression of IL-6, IL-10, and TNF-α in gingival tissues was similar in individuals with and without DM-2^10^. Collin, et al.^6^ (2000) observed similar salivary levels of MMP-9 between diabetic and non-diabetic individuals. Moreover, periodontal therapy has demonstrated the ability to reduce blood levels of CRP, as well as inflammatory cytokines such as IL-6 and TNF-α^23^.

Furthermore, we should consider the difficulty in quantifying the inflammatory process produced by periodontitis. The analysis of inflammatory mediators present in the saliva provides a global measure of oral inflammation. Although inflammatory cells present in the saliva mainly derive from the gingival crevicular fluid, cells from other inflammatory processes of the oral cavity may be present, contributing to the overestimated inflammation from periodontitis. On the other hand, the dilution by the saliva may underestimate the periodontal inflammation^30^.

Interestingly, in the current study, blood levels of CRP had significant impact on the DM-2 development among women with a history of GDM. Another inflammatory process could be involved in these CRP levels, as well as other mediators of inflammation, contributing to the manifestation of DM-2. This fact may suggest the role of a systemic inflammatory process in the development of insulin resistance. Systemic inflammation is significantly elevated in individuals with DM, including high levels of CRP^17^ ^,^ ^24^. A recent systematic review and meta-analysis^26^ examined the association between the inflammatory markers IL-6 and CRP and the risk of DM-2. Results showed a significant association between high levels of IL-6 and CRP and the risk of DM-2, suggesting the inflammatory process as predictor of the DM-2 development^26^. Similarly, women with GDM presented increased levels of CRP^5^ ^,^ ^9^. Furthermore, periodontitis has been associated with high CRP plasma levels^14^. This fact can support the biological plausibility of the impact of periodontal inflammation on DM-2. Therefore, different degrees of periodontitis may have different systemic repercussions.

The incidence of DM-2 among women with previous GDM was 18.4%. One study^16^ showed that 38.8% of women with GDM presented pre-diabetes and 6.6% presented DM-2 in a period of 5.5 years. The systematic review by Kim, Newton and Knopp^19^ (2002) showed a great variation in the incidence of DM-2 among women with history of GDM, from 2.6% to 70%. This great variability could be explained by differences in the follow-up period, in the diagnostic criteria for GDM, and in the sample selection.

Elevated BMI was significantly more frequent among women with altered glycemic levels, either diagnosed with pre-diabetes or DM-2, although it not remained significant in the multivariate final model. Obesity has been considered a risk factor for DM-2 development^2^. Studies investigating the association between periodontitis and GDM have demonstrated an elevated BMI associated with GDM^4^ ^,^ ^5^ ^,^ ^9^ ^,^ ^13^ ^,^ ^28^. Moreover, obesity has been associated with periodontitis^21^.

In the present study, increased age showed no impact on the development of DM-2 among women with GDM. Increased age has been considered a risk factor for GDM^13^. In addition, it was also demonstrated that the prevalence of DM-2 increased with age^2^. The history of GDM common to all individuals included in the present study may be a possible explanation for age not showing a significant difference between groups. Sample size could be considered a limitation of the present study that may have contributed to this finding.

Loss of participants due to non-location, non-answer, or non-adherence could also be considered a limitation of the present study. From the 90 women enrolled in the initial sample, 49 completed the present study. The smaller sample due to the loss is a limitation; however, longitudinally, the results may be an important point of initial information on this issue, and thus directing future studies.

The period of three years follow-up after delivery for women with GDM is compatible according to the American Diabetes Association^2^. However, future studies may include longer periods of monitoring to check this possible influence.

In the present study, there was not any impact of periodontitis on the DM-2 development among women with previous GDM. However, the number of studies on this subject is reduced. It is possible that, in other populations with different periodontal conditions, periodontitis may demonstrate impact on DM-2 development among women with previous GDM. Therefore, additional studies on different populations are necessary to better understand the relationship between these two conditions.

The improvement of the knowledge about GDM is important, since this condition can be considered a unique opportunity for a preventive intervention in relation to DM-2, a condition with high morbidity and mortality. The incidence of DM-2 among women with previous GDM observed in the present study was high, what justifies the efforts directed towards the identification of the potential associated risk factors.

## Conclusion

The observed impact of CRP on DM-2 development among women with a history of GDM demonstrates that during the prenatal monitoring period, it is necessary to emphasize the multidisciplinary approach for the diagnosis and treatment of systemic inflammatory processes, minimizing the risk for developing an insulin resistance. Uncertainties about the influence of periodontitis in the development of DM-2 among women with previous GDM do exist, signaling the need for additional studies.
